# SIRT1 and antioxidants in infertile females: Exploration of the role of vitamin D

**DOI:** 10.1371/journal.pone.0287727

**Published:** 2023-07-10

**Authors:** Faiza Alam, Maheen Shahid, Sumaira Riffat, Ihsan Nazurah Zulkipli, Fatima Syed, Mussarat Ashraf, Rehana Rehman

**Affiliations:** 1 PAPRSB Institute of Health Sciences, Universiti Brunei Darussalam, Bandar Seri Begawan, Brunei Darussalam; 2 Department of Biological and Biomedical Sciences, Aga Khan University, Karachi, Pakistan; 3 Jinnah Sindh Medical University, Karachi, Pakistan; 4 Fatima Syed, Fazaia Ruth Pfau Medical College–FRPMC, Karachi, Pakistan; University of Rijeka Faculty of Medicine: Sveuciliste u Rijeci Medicinski fakultet, CROATIA

## Abstract

**Background:**

Deficiency of silent information regulator 1 (SIRT1) can trigger inflammation, mitochondrial malfunctioning, and apoptosis through the hypothalamic-pituitary-ovarian axis, producing poor quality oocytes, leading to infertility. Normal vitamin D (VD) levels promote SIRT1 activity required for optimal fertility, and low levels of either may result in fertility problems owing to cell-membrane de-stabilization, increased autophagy, DNA damage leading to increased reactive oxygen species and mitochondrial dysfunction. Therefore, in this study, we want to estimate the levels of VD, SIRT1 and antioxidants (MnSOD; manganese superoxide dismutase, GR; glutathione reductase, visfatin) and oxidants (adrenaline & cortisol) in individuals living with infertility and explore the association of VD with SIRT1 expression (levels), antioxidants, and oxidants contributing to infertility in women. The significance of this study is that it highlights the importance of maintaining optimal levels of VD for reproductive health in females.

**Methods:**

This cross-sectional study included 342 (135 infertile and 207 fertile) female subjects. Serum levels of MnSOD, SIRT1, visfatin, GR, VD, adrenaline, and cortisol were analyzed by ELISA and were compared in fertile and infertile samples using the Mann Whitney U test.

**Results:**

There were significantly high levels of VD, SIRT1, GR, MnSOD and visfatin in fertile female participants. However, mean adrenaline and cortisol levels were higher in infertile samples with a significant negative correlation with VD. A significant negative correlation of VD with MnSOD, SIRT1, visfatin and GR was observed (p <0.01). In VD subset groups, MnSOD levels were significantly high in VD sufficient groups however, adrenaline and cortisol levels were significantly high in groups suffering from VD deficiency.

**Conclusions:**

Deficiency of VD is associated with a decrease in SIRT1 and other antioxidants, which may deter natural reproductive functions leading to infertility. Further studies are required to determine the cause-effect relationship of VD deficiency on conception and interpretation of the involved mechanism.

## Introduction

Infertility is defined as the inability for a couple to conceive after 12 months of regular sexual intercourse [1]. In the reproductive cycle, follicular granulosa cells form an important association with follicle’s survival that determines ovulation and hence fertility [2]. The development of follicles in the available follicle pool is dependent on a number of factors like hypoxia, heat stress, and oxidative stress (OS) [3].

Several toxicants and pesticides modify the defence system producing various lifestyle-related diseases due to OS and reproductive disorders like endometriosis, PCOS, preeclampsia, spontaneous abortion, and unexplained infertility [4, 5]. High levels of OS markers are hypothesized to compromise the quality of oocyte and subsequently, reproductive potential in women living with infertility [6]. Therefore, it is essential that the redox environment in the oocyte is maintained at beneficial levels.

**Fig 1:**
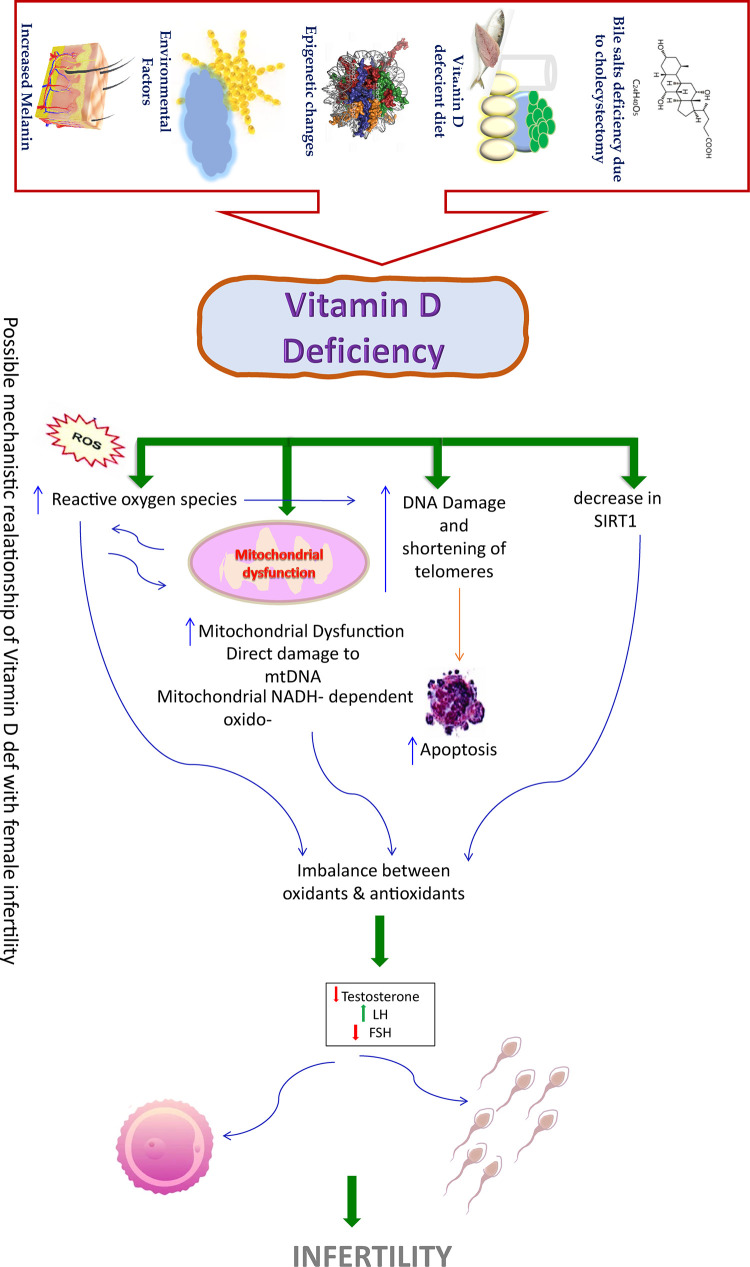
Hypothetical mechanisms predicting interplay of vitamin D and SIRT1 deficiency mitochondrial dysfunction leading to infertility. SIRT1, silent information regulator 1; ROS, reactive oxygen species; FSH, follicle-stimulating hormone; LH, luteinizing hormone.

VD acts as a membrane antioxidant, protects cell membranes against free radical-induced lipid peroxidation through interface with phospholipid fatty acid side chains and intensifies stabilization of the membrane structure and follicular development [7–9]. VD status is considered to be deficient, inefficient and sufficient on the basis of serum 25-hydroxyvitamin D (25(OH)D) level of less than 20 ng/mL, between 20 to 29.9 ng/ml greater than 30 ng/ml30 ng/mL [10]. VD levels above 100 ng/ml are labelled hypervitaminosis D [11]. Deficiency of VD with an increase in OS markers, altered sperm parameters and impaired fertility has already been explored [12]. VD supplementation in women has been shown to reduce the accumulation of pro-inflammatory advanced glycation end-products (AGEs) and inhibit the formation of reactive oxygen species (ROS) in ovarian tissue [8]. The estimation in follicular fluid (FF) of women living with infertility has suggested its important role in adjusting steroid hormones with a reduction in OS and hence enhancing chances of fertility [13]. (Fig 1).

Silent information regulator 1 (SIRT1) is a histone deacetylase one of the members of the Sirtuins family, and has been shown to maintain the redox environment in the body, *via* the regulation of mitochondrial OS [14]. Mitochondrial dysfunction, which may also affect the redox environment in the cell, has been linked to infertility, *via* the Sirtuins family of proteins [15]. It works through transcription factor Forkhead box O3 (FOXO3a) and peroxisome-proliferator activator-receptor γ-coactivator 1α (PGC-1α), which is a transcription co-activator. Together, they induce the expression of antioxidant genes and deacetylation of histone proteins [16].

SIRT1 regulates many antioxidants, including manganese superoxide dismutase (MnSOD) [16]. It is a mitochondrial antioxidant, playing an essential role in the aerobic respiration of cells [6]. SIRT1, known to regulate many antioxidant levels, is positively correlated with visfatin, glutathione reductase (GR), adrenaline, and VD. These findings represent the positive influence of elevated Sirtuins on a total antioxidant status and a negative association with cortisol [17].

Glutathione is a family of enzymes that includes glutathione peroxidase (GPx), glutathione S-transferase (GST), and glutathione reductase (GSH) [18]. It is an endogenous antioxidant that protects the cell from OS and also preserves other antioxidants such as vitamins C and E; the role of these vitamins has been already documented [19]. Visfatin is an adipokine released from visceral adipose tissue dominantly [20]. Superoxide dismutase 1 (SOD1) knockout mice show that SOD1 is required for the development of ovaries and female reproductive function [21].

Adrenaline is a chemical stressor released from the adrenal medulla as well as cortisol. Cortisol does affect the maternal metabolic adaptions and embryonic development [22]. Increased levels of SIRT1 are related to decreased levels of cortisol [23]. Therefore, in this study, we want to estimate the levels of VD, SIRT1 and antioxidants (MnSOD, GR and visfatin) and oxidants (adrenaline & cortisol) in individuals living with infertility and explore the association of VD with SIRT1 expression (levels), antioxidants, and oxidants contributing to infertility in women.

## Materials & methods

### Ethics statement

This study received extended approval from the Ethical Review Committee (ERC: 2020-0314-14433), Aga Khan University, Karachi, Pakistan.

### Study design, duration & setting

The cross-sectional study was carried out from July 2020 to July 2021 at the Aga Khan University (AKU), Karachi, Pakistan collaborating with the Australian Concept of Infertility Medical Centre (ACIMC), Karachi, Pakistan.

### Subject selection

Patients were recruited from the infertility clinic (ACIMC) during the clinic visits employing the convenient sampling method. The sample size was estimated using Open-Source Epidemiologic Statistics for Public Health. Observing Pakistan’s infertility rate of 23% and in order to achieve 80% power and detecting an odds ratio of at least two, assuming hypothetical proportion of control with exposure to be 23%, and two-sided confidence (1-alpha) of 5%, the minimum sample size was 320 female participants [24]. Out of the sample size of 342 women, there were 135 (39.5%) and 207 (60.5%) fertile female subjects. All patients were taken after acquiring written informed consent. Patients were examined for general health checkups, height and weight measurements, and body mass index (BMI) calculations were done. The baseline hormonal profile (follicular stimulating hormone, luteinizing hormone) was obtained and noted from desk records of ACIMC.

### Inclusion criteria

Fertile women aged 18–40 years old were included in the study from all ethnic backgrounds bearing a child less than three years of age and were in sexual interaction with their male partners for at least the preceding three months. The cases comprised female subjects with primary infertility of more than two years.

### Exclusion criteria

Women with secondary infertility, including ovarian cysts and endometriosis with ovarian pathologies, who had been using contraceptive pills in the preceding three months were excluded from the study. Women with endocrine disorders like diabetes mellitus and thyroid problems were also excluded.

### Blood sample collection

From each subject, 3 ml blood samples were collected in a tube. The collected samples were transported in ice boxes to AKU Multidisciplinary laboratory for storage and further analysis. The cases were previously diagnosed cases of infertility.

### Enzyme-Linked Immunosorbent Assay (ELISA)

Serum samples were used for estimation of MnSOD, SIRT1, visfatin, glutathione reductase, VD, adrenaline, and cortisol in fertile and infertile female participants.

- **Estimation of MnSOD**. Serum manganese superoxide dismutase was estimated with the commercially available Enzyme Immunoassay kit for Human Mitochondrial Superoxide dismutase (SOD2) ELISA Kit, Cat. No. SG-10189 (Sinogeneclon Co., Ltd., Hangzhou, China) according to the manufacturer’s protocol. The sensitivity of the kit was 3 pg/ml and the detection range of the standard was 15.6–1000 pg/ml with intra and inter-assay coefficient of variation: CV<8% and CV<10% respectively.
- **Estimation of SIRT1**. Serum SIRT1 were analyzed with a commercially available Enzyme Immunoassay kit for Human Sirtuin 1 (SIRT1) ELISA Kit, Cat. No. SG-10458 (Sinogeneclon Co., Ltd., HangZhou, China) according to manufacturer’s protocol. The sensitivity of the kit was 0.1 ng/ml and detection range of the standard was 0.5–18 ng/ml with intra and inter assay coefficient of variation: CV<8% and CV<10% respectively.
- **Estimation of visfatin**. Serum visfatin was determined by Human visfatin (VF) ELISA Kit, Cat. No. SG-10381 (Sinogeneclon Co., Ltd., HangZhou,China) according to manufacturer’s protocol. The sensitivity of the kit was 0.3 μg/L and detection range of the standard was 1–20 μg/L with intra and inter assay coefficient of variation: CV<8% and CV<10% respectively.
- **Estimation of glutathione reductase**. Serum glutathione reductase was determined by Human Glutathione Reductase (GR) ELISA Kit, Cat. No. SG-00523 (Sinogeneclon Co., Ltd., HangZhou, China) according to manufacturer’s protocol. The sensitivity of the kit was 0.1 pg/ml and detection range of the standard was 18–1000 pg/ml with intra and inter assay coefficient of variation; CV<8% and CV<10% respectively.
- **Estimation of vitamin D**. VD was analyzed by a 25(OH) Vitamin D ELISA kit, Cat. No. ab213966 (Abcam, Waltham, MA 02453, USA) according to manufacturer’s protocol. The sensitivity of the kit was 1.98 ng/ml and detection range of the standards was 0.5–1010 ng/ml with intra and inter assay coefficient of variation: CV<4% and CV<15% respectively.
- **Estimation of adrenaline**. Serum adrenaline was analyzed with a Human Epinephrine (EPI) ELISA Kit, Cat. No. SG-10545 (Sinogeneclon Co., Ltd., HangZhou, China) according to manufacturer’s instructions. The sensitivity of the kit was 0.25 ng/ml and the detection range of the standards was 7–150 ng/L with intra and inter-assay coefficient of variation: CV<8% and CV<10% respectively.
- **Estimation of cortisol**. Serum cortisol was determined using a Cortisol ELISA Kit, Cat. No. DKO001 (DiaMetra, Perugia, Italy) as per manufacturer’s instruction. The assay range of the kit was 10–500 ng/mL.

### Quantitative variables

The quantitative variables were divided into two categories: women who were fertile and infertile. The chosen grouping categories were based on causes of infertility including PCOS, male factor, endometriosis, tubal and unexplained infertility. These groups were chosen based on the increasing number of women attending infertility clinic with these causes.

### Statistical analysis

Data were analyzed by using IBM SPSS Statistics (RRID: SCR_016479) version 23.0. Counts with percentages have been reported for baseline features of studied samples.

To determine the distribution of the variable studied parameters of fertility, it was important for choosing an appropriate statistical method. Thus, the Shapiro-Wilk test was performed and showed that the distribution of all parameters departed significantly from normality (p-value < 0.01). Based on this outcome, non-parametric tests (Mann-Whitney U, Spearman’s correlation) were used, and the median with the interquartile range was used to summarize the variables. Mann-Whitney U test was performed to compare the differences in fertility parameters between the fertile and infertile groups. Spearman’s correlation analysis was performed to study the strength of the association between parameters.

## Results

Table 1 reports the baseline characteristics of the participants, in the present study, there were 342 samples; primary type of infertility was higher than the secondary cause. With median age of 22 (19–26) years at the time of marriage, the duration of marriage was 7(4–11) years. Results showed that the highest cause of infertility was due to PCOS followed by endometriosis.

**Table 1:** Baseline characteristics of studied subjects.

Variables of overall subjects (n = 342)	Median (IQR)	
Age	31 (27–36)	
BMI <21 kg/m^2^	20.45 (19.25–20.67)	
BMI 21–24.99 kg/m^2^	23.05 (22.04–23.79)	
BMI ≥25 kg/m^2^	27.73 (25.74–29.75)	
Baseline FSH	6 (5–7)	
Baseline LH	6 (4–8)	
Age of Marriage	22 (19–26)	
Duration of Marriage	7 (4–11)	
**Characteristics of infertile subjects** (n = 135)	***n* (%)**	
Type of Infertility	Primary	100 (74.1)
	Secondary	35 (25.9)
Cause of Infertility	PCOS	59 (43.7)
	Male Factor	11 (8.1)
	Endometriosis	14 (10.4)
	Tubal	11 (8.1)
	Unexplained	40 (29.6)

PCOS, polycystic ovary syndrome; BMI, body mass index; FSH, follicle-stimulating hormone; LH, luteinizing hormone.

**Fig 2:**
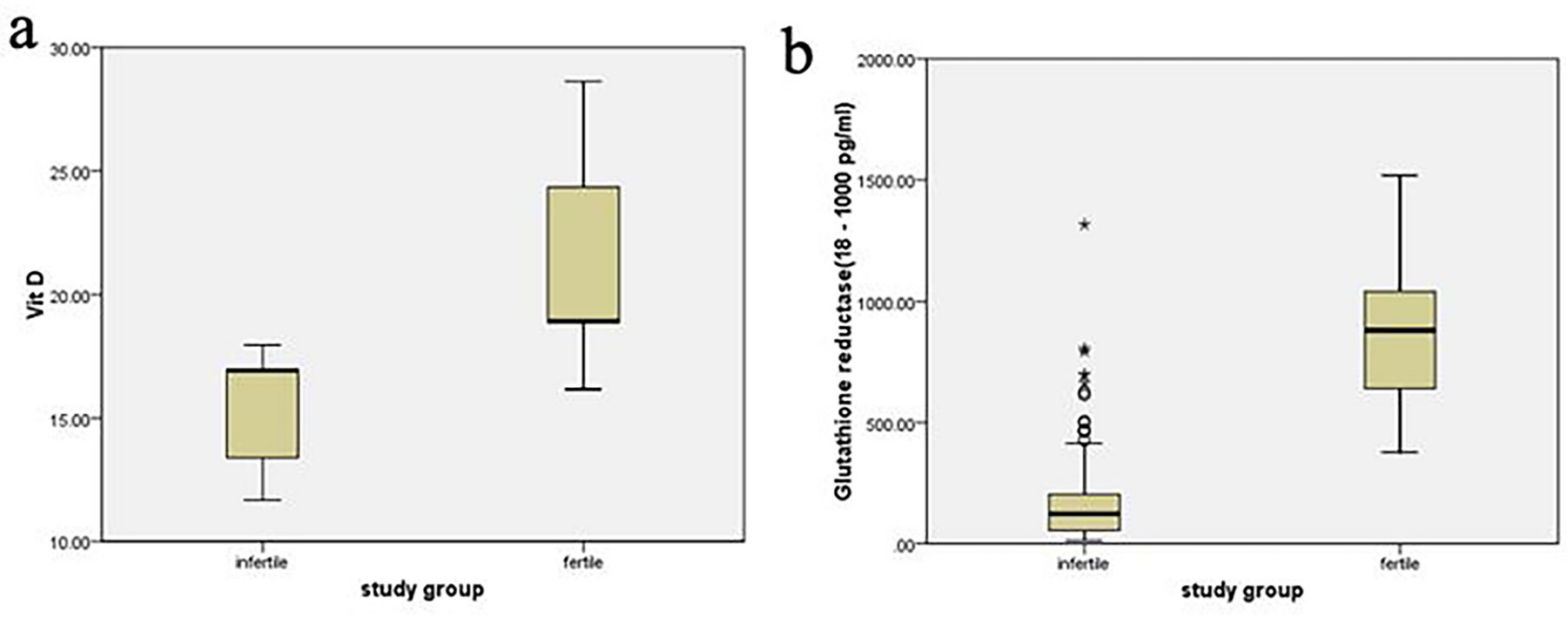
Comparison of levels of study variables when stratified based on fertility. (a) Vitamin D; (b) Glutathione Reductase.

Table 2 demonstrates the interquartile range of fertility parameters. All the antioxidants and levels of VD are statistically significantly higher in the fertile group; however, adrenaline and cortisol were lower in the fertile group when compared with the group living with infertility. Fig 2 represents the levels of VD and glutathione reductase in fertile controls and women with infertility.

**Table 2:** Comparison of oxidative markers in the controls and women living with infertility.

Parameters Median (IQR)	Infertile (n = 135)	Fertile (n = 207)	p-value
SIRT1 (ng/ml)	1.66 (0.9–5)	4.5 (2.4–5)	<0.01
Visfatin (ug/L)	4.26 (1.77–8)	6 (5–8)	<0.01
Glutathione reductase (pg/ml)	122.85 (53.37–202.09)	880 (640–1040)	<0.01
Vitamin D (ng/ml)	16.92 (13.39–16.92)	18.92 (18.92–24.35)	<0.01
MnSOD (ng/ml)	0.59 (0.44–0.94)	1.85 (0.48–3.2)	<0.01
Adrenaline	8.5 (5.1–47.54)	6.3 (4.8–9.8)	0.065
Cortisol	14.6 (9.02–20.59)	10.64 (9.06–12)	<0.01

SIRT1, silent information regulator 1; MnSOD, manganese superoxide dismutase; IQR, interquartile range.

Table 3 represents the correlation of VD with the study variables. The absolute magnitude of the coefficient (r) indicating the strength of the linear relationship between VD and MnSOD, SIRT1, Visfatin and the adrenaline was weak. The correlation of VD with Glutathione reductase was moderately strong (r = 0.507, p < 0.01).

**Table 3:** Correlation analysis of all study variables.

Correlations		
Parameters	Vitamin D	
	*Correlation Co-efficient* *r Value*	*p value*
Manganese SOD (0.15–10 ng/ml)	0.292**	<0.01
SIRT1 (0.5–18 ng/ml)	0.235**	<0.01
Visfatin (1–20 ug/L)	0.145*	0.007
Glutathione reductase (18–1,000 pg/ml)	0.507**	<0.01
Adrenaline	-0.239**	<0.01
Cortisol	-0.287**	<0.01

**Spearman Correlation is significant at the 0.05 level (2-tailed).

SOD, superoxide dismutase; SIRT1, silent information regulator 1.

Table 4 represents the levels of all study parameters based on VD deficient and sufficient groups. MnSOD levels were significantly high in VD sufficient groups however, adrenaline and cortisol levels were significantly high in groups suffering from VD deficiency.

**Table 4:** Serum levels of study parameters in vitamin D deficient and sufficient subsets.

Variables	Vitamin D Median (percentile 25–75)		*P value*
	*Deficient (n = 259)*	*Sufficient (n = 83)*	
***MnSOD***	0.67 (0.45–1.85)	1.85 (0.52–3.6)	<0.01
***SIRT1***	3 (0.97–5.0)	4.2 (3.0–5.0)	0.21
***Visfatin***	6.0(3.21–8.0)	5.0 (5.0–8.0)	0.268
***Glutathione reductase***	587.12 (116–880)	587.12 (480–800)	0.11
***Adrenaline***	8.10 (4.8–45.75)	5.9 (4.9–6.9)	<0.01
***Cortisol***	12 (9.0–18.14)	10.17 (9.35–10.69)	<0.01

Dependent Variable: Vitamin D

Mann-Whitney U test

Deficient: <20ng/ml

Sufficient: >30ng/ml

Insufficient: 20.1–29.9ng/ml (no sample was categorized as Vitamin D insufficient

None of the study subjects fell in the insufficient group of VD.

## Discussion

Maintenance of redox balance is essential for healthy reproduction. In this study, samples from 342 women living with infertility belonging to different ethnicities residing in Pakistan were taken; their age, duration of the marriage, age at the time of marriage, cause of infertility, BMI, and baseline follicle-stimulating hormone (FSH) and luteinizing hormone (LH) were noted; and the relation of oxidant and antioxidant in both fertile and infertile women. The chances of infertility were increased with greater proportion of oxidants, observed with increase in BMI and progression of age [17].

Advancing age is well-known to reduce the ovarian reserve and hence the reproductive potential of a woman [25]. According to the free radical theory of aging, it is the consistent accumulation of ROS and reduced antioxidant capacity that supplements the damages of aging [26]. Velthut *et al*., in their study reported reduced developmental potential seen during *in vitro* fertilization (IVF)/ intracytoplasmic sperm injection (ICSI) pregnancy in samples with low antioxidant levels [27].

Animal studies have shown that SIRT1-null mice have disrupted cycles and negatively impacted embryogenesis. Offspring of SIRT1-null mice were smaller in size than standard offspring and they mostly died in the early postnatal period only [25] SIRT1 downregulation in animal models is associated with follicle loss [25] and SIRT1 upregulation is noted to increase the fertilization rate. Diets causing SIRT1 suppression negatively impact fertilization [28]. A similar pattern is recorded in our sample, where SIRT1 was significantly lower in female participants living with infertility compared with fertile controls. Our data showed that PCOS was the cause of infertility in 43.7% of female subjects and endometriosis in 10.4%. Sirtuins improve fertility in women with PCOS by reducing androgen production by both ovaries and adrenals and improving insulin sensitivity [29] SIRT1, therefore, plays a vital role in oocyte physiology.

The results of our study showed low levels of MnSOD in women living with infertility compared with the controls. These results are in line with another study by Alam *et al*., where low levels of MnSOD were observed in women living with infertility with SIRT polymorphism, creating a microenvironment for the oocyte with oxidative stress [6]. Pournourali *et al*., in Iran, studied the relationship between MnSOD polymorphisms and female infertility and reported a positive association [30]. However, a previous study revealed that high levels of MnSOD activity may hinder average growth and fertility [31].

Animal studies have shown that increasing glutathione concentration reduces ROS and improves embryo growth during *in vitro* maturation (IVM) [32]. This is in line with our findings, where the levels of GR were significantly lower in subjects with infertility than in fertile controls. GR was positively associated with both VD and adrenaline and negatively associated with cortisol. In another study, lower GR and high cortisol, similar to that described above, were noted in women living with infertility and slow IVF responders, representing that low GR may cause reduced ovarian responsiveness to treatment [17].

Chan *et al*., in their study, reported higher visfatin levels in women with PCOS compared with women of similar BMI [33]. It is noteworthy that of our study subjects living with infertility, 43.7% was due to PCOS. Our study sample demonstrated a similar pattern of higher visfatin in subjects living with infertility compared with fertile controls. The literature has shown reduced visfatin levels in older female mice, and administration of visfatin has been shown to improve oocyte competence and reproductive potential by improving ovarian angiogenesis, [34]. Hussein *et al*., also found a positive association between high visfatin levels and female infertility, especially in PCOS, when associated with insulin resistance [35].

Stress-related cortisol release results in altered hormonal signaling, which influences the hypothalamic pituitary adrenal (HPA) axis as well as the hypothalamic-pituitary-ovarian (HPO) axis, thereby reducing female fertility by altering LH and luteinizing hormone-releasing hormone (LHRH) levels [36, 37]. Csemiczky *et al*., in their study, found significantly high cortisol levels in women living with infertility starting IVF treatment for tubal infertility compared to fertile control subjects; this difference in cortisol level was noted throughout the menstrual cycle [38]. our study sample also showed a similar pattern of significantly high cortisol in subjects living with infertility compared with fertile control subjects.

In the process of continuous exploration of the effects of OS on fertility, we found that MnSOD, a mitochondrial antioxidant, was positively correlated with SIRT1, visfatin, GR, and VD, and was negatively associated with adrenaline and cortisol. This shows that in cases of high stress (represented by high adrenaline and cortisol), antioxidant levels are reduced, which exacerbates the damages caused by it [23].

Burt *et al*., discovered that VD level is negatively associated with metanephrine, a breakdown product of catecholamine (includes dopamine, adrenaline, and noradrenaline) [39]. Respective studies reported adrenaline demonstrating seasonal variation with a higher catecholamine concentration in winters; these findings suggest a potential role of VD in fluctuating adrenaline levels [40, 41]. A study on chronically stressed rats secondary to OS, reports reduced glutathione and SOD levels, similar to what was noted in the present study; VD supplementation in these rats resulted in a rise in glutathione and SOD levels along with a reduction in adrenaline and noradrenaline levels.

It is documented that both VD and SIRT1 activity is required for optimal fertility, and low levels of either may result in fertility problems. Both are intrinsically linked in the cell, as i*n vitro* results show that VD treatment on adipocyte cells increased cellular SIRT1 activity and the NAD-NADH ratio [42]. VD supplementation resulted in a rise in antioxidants and a decline in oxidant levels, suggesting the protective role of VD against OS in animal models [43]. In human studies, VD supplementation improved T2D by decreasing HbA1c and increasing SIRT1 and irisin in VD deficient Type 2 Diabetic patients [44].

Our study has documented significantly high levels of antioxidant MnSOD in the VD sufficient group predicting a causal relationship between both variables. However, clinical trials are suggested to prove the cause of the relationship.

### Limitations

This study had a few limitations including sampling, which was done during the clinic timings (9 am to 2 pm); however, estimation of cortisol should be done early in the morning around 6 am. Furthermore, variation of adrenaline levels after vein puncture or even after seeing the needle is expected and could not be overruled in our study.

## Conclusions

Oxidants and antioxidants impact female fertility. The deficiency of VD is associated with a decrease in SIRT1 and other antioxidants, which may deter natural reproductive functions leading to infertility. The study suggests that VD deficiency can result in decreased levels of antioxidants and SIRT1, leading to inflammation, mitochondrial malfunctioning, and apoptosis through the hypothalamic-pituitary-ovarian axis, resulting in poor-quality oocytes and infertility. The study findings further propose that VD supplementation may be a potential therapeutic option for improving female fertility. However, further studies are required to determine the cause-effect relationship of VD deficiency on conception and the involved mechanisms. Overall, this study provides valuable insights into the potential role of VD and antioxidants in female infertility.

### Wider implications

SIRT1 is a notable target in various disease situations due to the promise of pharmacological and/or natural modulators of SIRT1 activity within the framework of endocrine and immune-related disease models.

## Supporting information

S1 File (XLS)

S1 Graphical abstract (TIF)

## References

[1] Vander Borght M, Wyns C. Fertility and infertility: Definition and epidemiology. Clin Biochem. 2018;62:2–10. doi: 10.1016/j.clinbiochem.2018.03.012 29555319

[2] Bhardwaj JK, Saraf P. Ameliorating potentials of N-acetyl-l-cysteine against methoxychlor instigated modulation in structural characteristics of granulosa cells of caprine antral follicles. Indian J. Biochem. Biophys. 2021., 58, 365–371

[3] Bhardwaj JK, Paliwal A, Saraf P, Sachdeva SN. Role of autophagy in follicular development and maintenance of primordial follicular pool in the ovary. J Cell Physiol. 2022;237(2):1157–70. doi: 10.1002/jcp.30613 34668576

[4] Bhardwaj JK, Mittal M, Saraf P, Kumari P. Pesticides induced oxidative stress and female infertility: a review. Toxin Rev. 2020;39(1):1–13.

[5] Bhardwaj JK, Panchal H, Saraf P. Ameliorating effects of natural antioxidant compounds on female infertility: a review. Reprod Sci. 2021;28:1227–56. doi: 10.1007/s43032-020-00312-5 32935256

[6] Alam F, Rehman R, Fatima SS, Ashraf M, Khan TA. Suggested role of silent information regulator 1 (SIRT1) gene in female infertility: A cross‐sectional study in Pakistan. Int J Clin Pract. 2021;75(6):e14132. doi: 10.1111/ijcp.14132 33735475

[7] Kim HA, Perrelli A, Ragni A, Retta F, De Silva TM, Sobey CG, et al. Vitamin D deficiency and the risk of cerebrovascular disease. Antioxidants. 2020;9(4):327. doi: 10.3390/antiox9040327 32316584 PMC7222411

[8] Irani M, Merhi Z. Role of vitamin D in ovarian physiology and its implication in reproduction: a systematic review. Fertil Steril. 2014;102(2):460–8. e3. doi: 10.1016/j.fertnstert.2014.04.046 24933120

[9] Kinuta K, Tanaka H, Moriwake T, Aya K, Kato S, Seino Y. Vitamin D is an important factor in estrogen biosynthesis of both female and male gonads. Endocrinology. 2000;141(4):1317–24. doi: 10.1210/endo.141.4.7403 10746634

[10] Antonenko O, Bryk G, Brito G, Pellegrini G, Zeni S. Oral health in young women having a low calcium and vitamin D nutritional status. Clin Oral Investig. 2015;19:1199–206. doi: 10.1007/s00784-014-1343-x 25359326

[11] Özkan B, Hatun S, Bereket A. Vitamin D intoxication. Turk J Pediatr. 2012;54(2):93. 22734293

[12] Shahid M, Khan S, Ashraf M, Akram Mudassir H, Rehman R. Male infertility: Role of vitamin D and oxidative stress markers. Andrologia. 2021;53(8):e14147. doi: 10.1111/and.14147 34247390

[13] Masjedi F, Keshtgar S, Agah F, Karbalaei N. Association between sex steroids and oxidative status with vitamin D levels in follicular fluid of non-obese PCOS and healthy women. J Reprod Infertil. 2019;20(3):132. 31423416 PMC6670262

[14] Li L, Wang H, Zhao S, Zhao Y, Chen Y, Zhang J, et al. Paeoniflorin ameliorates lipopolysaccharide‐induced acute liver injury by inhibiting oxidative stress and inflammation via SIRT1/FOXO1a/SOD2 signaling in rats. Phytother Res. 2022. doi: 10.1002/ptr.7471 35570830

[15] Di Emidio G, Falone S, Artini PG, Amicarelli F, D’Alessandro AM, Tatone C. Mitochondrial sirtuins in reproduction. Antioxidants. 2021;10(7):1047. doi: 10.3390/antiox10071047 34209765 PMC8300669

[16] Olmos Y, Sánchez-Gómez FJ, Wild B, García-Quintans N, Cabezudo S, Lamas S, et al. SirT1 regulation of antioxidant genes is dependent on the formation of a FoxO3a/PGC-1α complex. Antioxid Redox Signal. 2013;19(13):1507–21. 23461683 10.1089/ars.2012.4713 PMC3797451

[17] Alam F, Khan TA, Amjad S, Rehman R. Association of oxidative stress with female infertility-A case control study. J Pak Med Assoc. 2019;69(5). 31105280

[18] Agarwal A, Aponte-Mellado A, Premkumar BJ, Shaman A, Gupta S. The effects of oxidative stress on female reproduction: a review. Reprod Biol Endocrinol. 2012;10(1):1–31. doi: 10.1186/1477-7827-10-49 22748101 PMC3527168

[19] Bhardwaj JK, Mittal M, Saraf P, Sharma S. Ameliorative potential of vitamin C and E against Roundup‐glyphosate induced genotoxicity triggering apoptosis in caprine granulosa cells. Environ Mol Mutagen. 2022;63(5):246–54. doi: 10.1002/em.22497 35770910

[20] Nataraj SK, Girija K. Biochemical Changes in Female Infertility: Highlights on Leptin, Adiponectin, Visfatin, and Resistin. Indian J Med Biochem. 2019;23(3):339–42.

[21] Matzuk MM, Dionne L, Guo Q, Kumar TR, Lebovitz RM. Ovarian function in superoxide dismutase 1 and 2 knockout mice. Endocrinology. 1998;139(9):4008–11. doi: 10.1210/endo.139.9.6289 9724058

[22] Whirledge S, Cidlowski JA. Glucocorticoids, stress, and fertility. Minerva Endocrinol. 2010;35(2):109. 20595939 PMC3547681

[23] Alam F, Khan TA, Ali R, Tariq F, Rehman R. SIRTI and cortisol in unexplained infertile females; a cross sectional study, in Karachi Pakistan. Taiwan J Obstet Gynec. 2020;59(2):189–94. doi: 10.1016/j.tjog.2020.01.004 32127136

[24] Kelsey JL, Thompson WD, Evans AS. Methods in Observational Epidemiology (Monographs in Epidemiology & Biostatistics): Oxford University Press.

[25] Tatone C, Di Emidio G, Vitti M, Di Carlo M, Santini S, D’Alessandro AM, et al. Sirtuin functions in female fertility: possible role in oxidative stress and aging. Oxid Med Cell longev. 2015;2015. doi: 10.1155/2015/659687 26075037 PMC4436464

[26] Harman D. Aging: a theory based on free radical and radiation chemistry. Sci. aging knowledge environ. 2002;2002(37):cp14–cp.

[27] Velthut A, Zilmer M, Zilmer K, Kaart T, Karro H, Salumets A. Elevated blood plasma antioxidant status is favourable for achieving IVF/ICSI pregnancy. Reprod Biomed Online. 2013;26(4):345–52. doi: 10.1016/j.rbmo.2012.12.012 23415995

[28] Wang N, Luo L-L, Xu J-J, Xu M-Y, Zhang X-M, Zhou X-L, et al. Obesity accelerates ovarian follicle development and follicle loss in rats. Metabolism. 2014;63(1):94–103. doi: 10.1016/j.metabol.2013.09.001 24135502

[29] Banaszewska B, Wrotyńska-Barczyńska J, Spaczynski RZ, Pawelczyk L, Duleba AJ. Effects of resveratrol on polycystic ovary syndrome: a double-blind, randomized, placebo-controlled trial. J Clin Endocrinol Metab. 2016;101(11):4322–8. doi: 10.1210/jc.2016-1858 27754722

[30] Pournourali M, Tarang A, Haghighi SF, Yousefi M, Bahadori MH. Polymorphism variant of MnSOD A16V and risk of female infertility in northern Iran. Taiwan J Obstet Gynec. 2016;55(6):801–3. doi: 10.1016/j.tjog.2016.06.018 28040123

[31] Raineri I, Carlson EJ, Gacayan R, Carra S, Oberley TD, Huang T-T, et al. Strain-dependent high-level expression of a transgene for manganese superoxide dismutase is associated with growth retardation and decreased fertility. Free Radic Biol Med. 2001;31(8):1018–30. doi: 10.1016/s0891-5849(01)00686-4 11595386

[32] Mukherjee A, Malik H, Saha AP, Dubey A, Singhal DK, Boateng S, et al. Resveratrol treatment during goat oocytes maturation enhances developmental competence of parthenogenetic and hand-made cloned blastocysts by modulating intracellular glutathione level and embryonic gene expression. J Assist Reprod Genet. 2014;31(2):229–39. doi: 10.1007/s10815-013-0116-9 24305840 PMC3933608

[33] Chan T-F, Chen Y-L, Chen H-H, Lee C-H, Jong S-B, Tsai E-M. Increased plasma visfatin concentrations in women with polycystic ovary syndrome. Fertil Steril. 2007;88(2):401–5. doi: 10.1016/j.fertnstert.2006.11.120 17335820

[34] Choi K-H, Joo B-S, Sun S-T, Park M-J, Son J-B, Joo J-K, et al. Administration of visfatin during superovulation improves developmental competency of oocytes and fertility potential in aged female mice. Fertil Steril. 2012;97(5):1234–41. e3. doi: 10.1016/j.fertnstert.2012.02.032 22425197

[35] Hussein H, Abdel Fadeil M, Ait-allah AS. Role of leptin and visfatin in infertility in obese and non obese women. Sohag Med J. 2018;22(1):65–71.

[36] Palomba S, Daolio J, Romeo S, Battaglia FA, Marci R, La Sala GB. Lifestyle and fertility: the influence of stress and quality of life on female fertility. Reprod Biol Endocrinol: RB&E. 2018;16(1):113. doi: 10.1186/s12958-018-0434-y 30501641 PMC6275085

[37] Cwikel J, Gidron Y, Sheiner E. Psychological interactions with infertility among women. Eur J Obstet Gynecol Reprod Biol. 2004;117(2):126–31. doi: 10.1016/j.ejogrb.2004.05.004 15541845

[38] Csemiczky G, Landgren BM, Collins A. The influence of stress and state anxiety on the outcome of IVF-treatment: psychological and endocrinological assessment of Swedish women entering IVF-treatment. Acta Obstet Gynecol Scandinavica. 2000;79(2):113–8. doi: 10.1034/j.1600-0412.2000.079002113.x 10696958

[39] Burt MG, Mangelsdorf BL, Stranks SN, Mangoni AA. Relationship between Vitamin D Status and Autonomic Nervous System Activity. Nutrients. 2016;8(9). doi: 10.3390/nu8090565 27649235 PMC5037550

[40] Kruse HJ, Wieczorek I, Hecker H, Creutzig A, Schellong SM. Seasonal variation of endothelin-1, angiotensin II, and plasma catecholamines and their relation to outside temperature. J Lab Clin Med. 2002;140(4):236–41. doi: 10.1067/mlc.2002.127169 12389021

[41] Pamporaki C, Bursztyn M, Reimann M, Ziemssen T, Bornstein SR, Sweep FC, et al. Seasonal variation in plasma free normetanephrine concentrations: implications for biochemical diagnosis of pheochromocytoma. Eur J Endocrinol. 2014;170(3):349–57. doi: 10.1530/EJE-13-0673 24497497

[42] Chang E, Kim Y. Vitamin D decreases adipocyte lipid storage and increases NAD-SIRT1 pathway in 3T3-L1 adipocytes. Nutrition. 2016;32(6):702–8. doi: 10.1016/j.nut.2015.12.032 26899162

[43] Hussien NI, El-Wakeel HS, Souror SM, Ahmed IA. Alleviation of cardiac mitochondrial dysfunction and oxidative stress underlies the protective effect of vitamin D in chronic stress-induced cardiac dysfunction in rats. Gen Physiol Biophys. 2019;38(1):51–61. doi: 10.4149/gpb_2018036 30761994

[44] Safarpour P, Daneshi-Maskooni M, Vafa M, Nourbakhsh M, Janani L, Maddah M, et al. Vitamin D supplementation improves SIRT1, Irisin, and glucose indices in overweight or obese type 2 diabetic patients: a double-blind randomized placebo-controlled clinical trial. BMC Fam Pract. 2020;21:1–10. 32033527 10.1186/s12875-020-1096-3 PMC7007689

