# Supplementary figures and images for: SIRT1 and antioxidants in infertile females: Exploration of the role of vitamin D
Source: PLoS One. 2023 Jul 10;18(7):e0287727. doi: 10.1371/journal.pone.0287727 (PMC10332578; doi:10.1371/journal.pone.0287727)

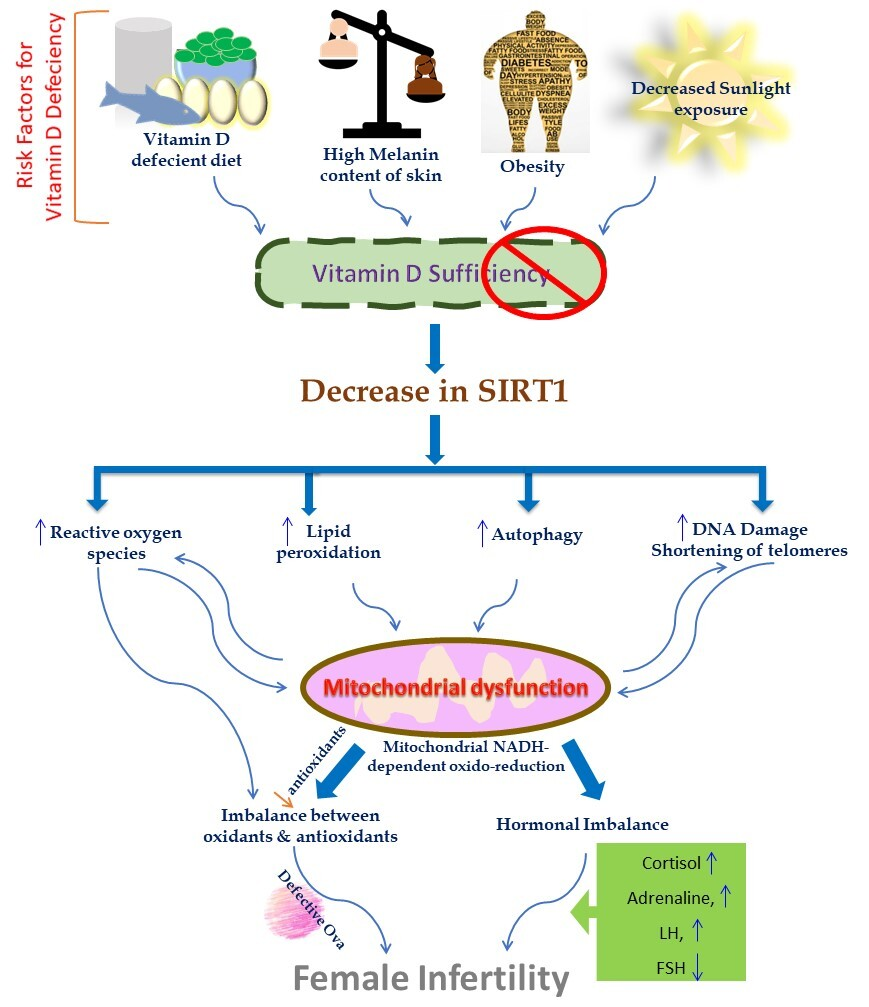

Supplement: S1 Graphical abstract — (TIF) [file pone.0287727.s002.tif]
